# Efficient Synergistic Single-Cell Genome Assembly

**DOI:** 10.3389/fbioe.2016.00042

**Published:** 2016-05-23

**Authors:** Narjes S. Movahedi, Mallory Embree, Harish Nagarajan, Karsten Zengler, Hamidreza Chitsaz

**Affiliations:** ^1^Department of Computer Science, Wayne State University, Detroit, MI, USA; ^2^Department of Bioengineering, University of California San Diego, San Diego, CA, USA; ^3^Department of Computer Science, Colorado State University, Fort Collins, CO, USA

**Keywords:** genome assembly, single-cell genomics, uncultivable bacteria, colored de Bruijn graph, genome coassembly

## Abstract

As the vast majority of all microbes are unculturable, single-cell sequencing has become a significant method to gain insight into microbial physiology. Single-cell sequencing methods, currently powered by multiple displacement genome amplification (MDA), have passed important milestones such as finishing and closing the genome of a prokaryote. However, the quality and reliability of genome assemblies from single cells are still unsatisfactory due to uneven coverage depth and the absence of scattered chunks of the genome in the final collection of reads caused by MDA bias. In this work, our new algorithm Hybrid *De novo* Assembler (HyDA) demonstrates the power of coassembly of multiple single-cell genomic data sets through significant improvement of the assembly quality in terms of predicted functional elements and length statistics. Coassemblies contain significantly more base pairs and protein coding genes, cover more subsystems, and consist of longer contigs compared to individual assemblies by the same algorithm as well as state-of-the-art single-cell assemblers SPAdes and IDBA-UD. Hybrid *De novo* Assembler (HyDA) is also able to avoid chimeric assemblies by detecting and separating shared and exclusive pieces of sequence for input data sets. By replacing one deep single-cell sequencing experiment with a few single-cell sequencing experiments of lower depth, the coassembly method can hedge against the risk of failure and loss of the sample, without significantly increasing sequencing cost. Application of the single-cell coassembler HyDA to the study of three uncultured members of an alkane-degrading methanogenic community validated the usefulness of the coassembly concept. HyDA is open source and publicly available at http://chitsazlab.org/software.html, and the raw reads are available at http://chitsazlab.org/research.html.

## 1 Introduction

Enormous progress toward DNA sequencing has brought a realm of exciting applications within reach, including genomic analysis at single-cell resolution. Single-cell genome sequencing holds great promise for various areas of biology including environmental biology (McLean et al., 2013). In particular, myriad unculturable environmental microorganisms have been studied using single-cell genome sequencing powered by high-throughput DNA amplification methods (Dean et al., 2001, 2002;Hosono et al., 2003; Gill et al., 2006; Rusch et al., 2007). Since the majority of microbes to date are unculturable, single-cell sequencing has enabled significant progress in elucidating the genome sequences and metabolic capabilities of these previously inaccessible microorganisms.

Single-cell sequencing, which was challenging and limited for years, is now accessible and attractive for many scientific fields according to the Nature Method of the year 2013. It helps various types of projects such as antibiotics discovery (Li and Vederas, 2009), Earth Microbiome Project (EMP) (Caporaso et al., 2012), and Human Microbiome Project (HMP) (Gill et al., 2006). The importance of single-cell sequencing is particularly due to the fact that only 1% of environmental bacteria have been cultured in the laboratory as they need their natural habitant for cultivation (Lasken, 2007). Also, single-cell sequencing can preserve the uniqueness of each cell and its individual mutations and structural variations, which are valuable information, especially in cancer studies.

Nevertheless, single-cell sequencing is still far from perfect as whole-genome amplification procedures are needed to augment femtograms of DNA material of one cell into micrograms. All known amplification reactions to date introduce some form of bias. Today, the dominant amplification method in single-cell sequencing technology is the Multiple Displacement Amplification (MDA) (Dean et al., 2001, 2002; Lasken and Egholm, 2003). Another popular amplification method is MALBAC, which causes its type of amplification artifact (Lu et al., 2012; Zong et al., 2012).

Multiple Displacement Amplification (MDA) is the preferred amplification method for single-cell sequencing, since it is an isothermal (without thermo cycling) process as opposed to PCR (Illumina, 2013, 2014). Compared to PCR-based amplification methods, it produces less amplification coverage bias and error (Tindall and Kunkel, 1988; Esteban et al., 1993; Pinard et al., 2006).

Recently, a new whole-genome amplification method has been demonstrated on individual human cells, which is called Multiple Annealing and Looping Based Amplification Cycles (MALBAC) (Lu et al., 2012; Zong et al., 2012). MALBAC coverage of the human genome has less bias than that of MDA. Nevertheless, amplification bias is still a challenge despite the improvements achieved by MALBAC (Daley and Smith, 2014). Furthermore, sensitivity of MALBAC to background noise makes it not suitable for many applications, such as *de novo* assembly (de Bourcy et al., 2014).

Although single-cell sequencing methods have passed important milestones, such as capturing ≥90% of genes in a prokaryotic cell (Chitsaz et al., 2011) or finishing and closing the genome of a prokaryote using MDA (Woyke et al., 2010), the quality and reliability of genome assemblies from single cells lag behind those of sequencing methods from multi cells due to a bias arising from MDA. The main factors that affect quality are uneven coverage depth and the absence of scattered chunks of the genome in the final collection of reads. There is no known deterministic pattern for the preferred amplified regions, and they are currently treated as the result of a random process. Also, the outcome of MDA is widely variable ranging from total loss of the sample and any information therein to nearly complete reconstruction of the genome. In this sense, an MDA-based single-cell sequencing experiment is currently a gamble that can potentially lead to the loss of the sample and sequencing expenses.

The uneven depth of coverage of a single-cell data set makes the result of *de novo* assembly with uniform sequencing depth assumption inaccurate (Rodrigue et al., 2009; Woyke et al., 2009). This makes the challenges of single-cell sequencing more computational than experimental (Rodrigue et al., 2009). A novel computational solution proposed by Chitsaz et al. (2011) overcomes some of the complications caused by uneven depth of coverage. That method is implemented into a tool called Velvet-SC and adapted by other subsequent single-cell assembly tools, such as SPAdes (Bankevich et al., 2012) and IDBA-UD (Peng et al., 2012), which introduce further advanced algorithmic features and outperform Velvet-SC.

No matter how sophisticated the algorithmic features of an assembler, there is no way to assemble those regions of the genome that are not amplified enough to be captured in sequencing. Chitsaz et al. (2011) called those absent parts of the genome *blackout regions*. We propose an elegant solution to retrieve those blackout regions using the information vested in other single-cell data sets. Coverage data of identical DNA molecules suggest that the MDA process has a strong random component to the extent that it is likely that the blackout regions in one reaction are fully covered in another one. We introduce a coassembly strategy, which can fill the blackout regions in a data set by using the information in another coassembled data set using the idea of colored de Bruijn graph (Iqbal et al., 2012).

Colored de Bruijn graph was initially introduced for structural variation detection. We modified and implemented the algorithm for single-cell coassembly. Furthermore, our algorithm modifies the iterative *k* assembly algorithm, which is implemented by SPAdes (Bankevich et al., 2012) and IDBA-UD (Peng et al., 2012), and adapts it to the colored graph (Shariat Razavi et al., 2014). It has been shown that the weakness of the coassembly is related to breaking contigs due to various colored branches (Movahedi et al., 2012). Iterative assembly with variable *k* overcomes that contiguity weakness.

We demonstrate in this work how to hedge against the risk of poor assembly results through sequencing and coassembly of few single cells. Our method replaces a single-cell deep sequencing experiment with multiple single-cell shallow sequencing experiments, allowing for the simultaneous acquisition of supposedly synergistic information about multiple single cells.

## 2 Materials and Methods

### 2.1 Media and Cultivation of the Methanogenic Alkane-Degrading Community

The microbial community was enriched from sediment from a hydrocarbon-contaminated ditch in Bremen, Germany (Zengler et al., 1999). The consortium was propagated in the laboratory in anoxic medium containing 0.3 g NH_4_Cl, 0.5 g MgSO_4_⋅7H_2_O, 2.5 g NaHCO_3_, 0.5 g K_2_HPO_4_, 0.05 g KBr, 0.02 g H_3_BO_3_, 0.02 g KI, 0.003 g Na_2_WO_2_⋅2H_2_O, 0.002 g NiCl_2_⋅6H_2_O, trace elements, and trace minerals as previously described (Zengler et al., 1999). The medium was sparged with a mixture of N_2_/CO_2_ (80:20 v/v), and the pH was adjusted to 7.0. After autoclaving, anoxic CaCl_2_ (final concentration 0.25 g/L) and filter-sterilized vitamin solution (Zengler et al., 1999) were added. Cells were supplemented with anoxic hexadecane as previously described (Embree et al., 2013). Bottles were degassed as necessary to relieve over-pressurization.

### 2.2 Single-Cell Sorting, MDA, and Genomes Sequencing

Individual cells from the alkane-degrading consortium were obtained by staining (SYTO-9 DNA stain) and sorting of single cells by FACS (Embree et al., 2013). Single cells were lysed as previously described, and the genomic DNA of individual cells was amplified using whole-genome multiple displacement amplification (MDA) (Swan et al., 2011). Amplified genomic DNA was screened for Smithella-specific 16S rDNA gene sequences. Six amplified Smithella genomes were selected for Next-Generation Sequencing. The MDA amplified genomes were prepared for Illumina sequencing using the Nextera kit, version 1 (Illumina) using the Nextera protocol (ver. June 2010) and high molecular weight buffer. Libraries with an average insert size of 400 bp were created for these samples and sequenced using an Illumina Genome Analyzer IIx. The 34-bp paired-end reads were generated for K05 (20.9 million reads), C04 (23.3 million reads), F02 (26.9 million reads), and A17 (22.2 million reads). The 58-bp single-end reads were generated for MEB10 (41.3 million reads), MEK03 (54.1 million reads), and MEL13 (18.0 million reads). The 36-bp paired-end reads were generated for F16 (11.0 million reads), K04 (27.2 million reads), and K19 (22.9 million reads).

### 2.3 Assembly of Single-Cell Genomes

Assemblies were obtained using HyDA version 1.1.1, SPAdes version 2.4.0, and IDBA-UD version 1.0.9. SPAdes and IDBA-UD were run with the default parameters in the single-end mode. The scripts to generate all of the assemblies are provided in Supplementary Material. The length of k-mers in the de Bruijn graph was 25, and the coverage cut off to trim erroneous branches in the graph was selected to be 100. The contigs were then annotated using RAST (Aziz et al., 2008), and the resulting annotation was used to generate a draft metabolic reconstruction using Model SEED (Henry et al., 2010). The Whole Genome Shotgun project has been deposited at DDBJ/EMBL/GenBank under the accession AWGX00000000. The version described here is version AWGX01000000.

## 3 Results

### 3.1 Colored de Bruijn Graph

Algorithmic paradigms for fragment assembly, such as overlap-layout-consensus and de Bruijn graph, depend on the characteristics of sequencing reads, particularly read length and error profile. Overlap-layout-consensus is a paradigm that is usually applied to assembly projects using long reads, and the de Bruijn graph is another widely adopted paradigm that is used for short-read data sets (Compeau et al., 2011). A number of consecutive *k*-mers (a sequence of length *k* nucleotides) replace each read in the de Bruijn graph paradigm. Each *k*-mer is represented by a unique vertex. An edge is present between two vertices if there is a read in which the two respective *k*-mers are consecutively overlapping. When there are at least *k* consecutive common bases, reads share a vertex (respectively, *k* + 1 common bases for an edge) along which contigs are efficiently constructed.

**Figure 1:**
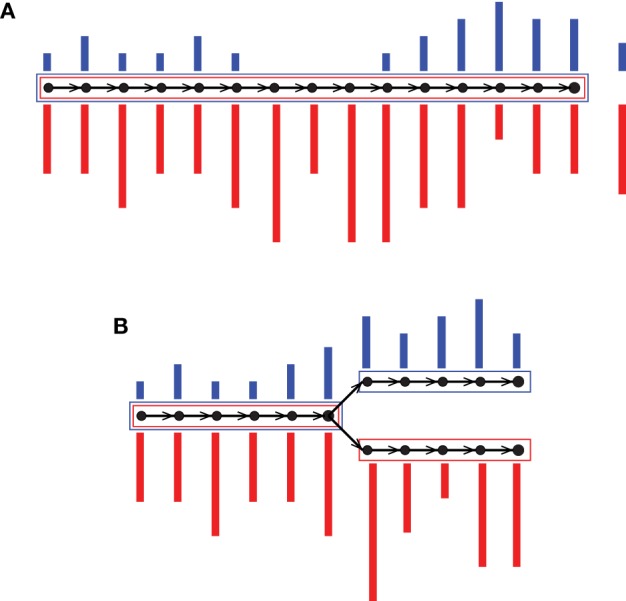
**Two sample colored de Bruijn graphs with colors red and blue**. Nodes are *k*-mers and edges represent *k* + 1-mers. A colored bar shows multiplicity of the *k*-mer in the corresponding colored input data set. Each box is an output contig, and the color of a box shows non-zero colored average coverage, which is shown on the right hand side of the contig in **(A)**. Our coassembly algorithm **(A)** rescues a poorly covered region of the genome in one color when it is well covered in the other, and **(B)** allows pairwise comparison of colored assemblies through revealing all of their shared and exclusive pieces of sequence.

Colored de Bruijn graph is a method proposed for coassembly of multiple short-read data sets (Iqbal et al., 2012). It is an extension of the classical approach by superimposing different uniquely colored input data sets on a single de Bruijn graph. Each vertex, which is a representation of a *k*-mer, accompanies an array of colored multiplicities. In this way, input data sets are virtually combined while they are almost fully tracked, enabling separation after assembly. Iqbal et al. (2012) proposed the colored de Bruijn graph in Cortex for variant calling and genotyping, whereas our tool Hybrid *De novo* Assembler (HyDA) (Movahedi et al., 2012) is developed for *de novo* assembly of short-read sequences with non-uniform coverage, which is a dominant phenomenon in MDA-based single-cell sequencing (Chitsaz et al., 2011). To fill the gaps and compare colors, contigs in HyDA are constructed in a color-oblivious manner, solely based on the branching structure of the graph. First, this method rescues a poorly covered region of the genome in one data set when it is well covered in at least one of the other input data sets (Figure 1A; Table 1). Second, it allows comparison of colored assemblies by revealing all shared and exclusive pieces of sequence not shorter than *k* (Figure 1B; Table 2).

**Table 1:** **The GAGE (Salzberg et al., 2012) statistics of HyDA assemblies for the six scenarios in Figure S1 in Supplementary Material**.

	Lane 1 Single color	Lane 6 Single color	Identical cells Mixed	Identical cells Colored	Non-identical cells Mixed	Non-identical cells Colored
Assembly size	4,532,221	4,642,640	5,262,077	5,204,061	8,273,488	5,212,674
Missing *E. coli* reference bases (%)	314,009 (6.77%)	123,687 (2.67%)	1,555 (0.03%)	2,023 (0.04%)	1,289 (0.03%)	2,136 (0.05%)
Extra bases (%)	280,998 (6.20%)	198,072 (4.27%)	653,307 (12.42%)	584,534 (11.23%)	3,661,052 (44.25%)	597,088 (11.45%)
SNPs	60	19	11	3	5	5
Indels < 5 bp	6	4	10	6	8	6
Indels ≥ 5 bp	13	14	6	5	4	4
Inversions	0	0	0	0	0	0
Relocations	12	11	2	3	2	3
NG50	42,257	54,422	41,964	34,752	54,505	37,794
Corrected NG50	39,975	44,872	39,334	32,876	39,334	36,868

*GAGE (Salzberg et al., 2012) was based on MUMmer 3.23 aligner (Kurtz et al., 2004)*.

**Table 2:** **Pairwise relationships between three coassembled data sets, *E. coli* lanes 1 and 6 and *S. aureus* lane 7, in a coassembly of *E. coli* lanes 1–4, 6–8, and *S. aureus* lanes 7 and 8**.

**Pair of data sets**	**Pair 1**		**Pair 2**		**Pair 3**	

	***E. coli* lane 1**	***E. coli* lane 6**		***S. aureus***		***E. coli* lane 1**

Total (bps)	5,228,480	5,240,302		3,366,622		5,228,480
Shared (bps)	5,210,548		335,648		336,184	
Exclusive (bps)	179,32	29,754	4,904,654	3,030,974	3,030,438	4,892,296
Exclusivity ratio^a^	0.003	0.005	0.9359	0.9003	0.9001	0.9357

*Total is the total size of those contigs that have non-zero coverage in the corresponding color. Shared is the size of those contigs that have non-zero coverage in both colors. Exclusive is the size of those contigs that have non-zero coverage in the corresponding color and zero coverage in the other color in the pair*.

*^a^Exclusivity ratio = exclusive/total*.

### 3.2 Coverage Characteristics of Single-Cell Read Data Sets

**Figure 2:**
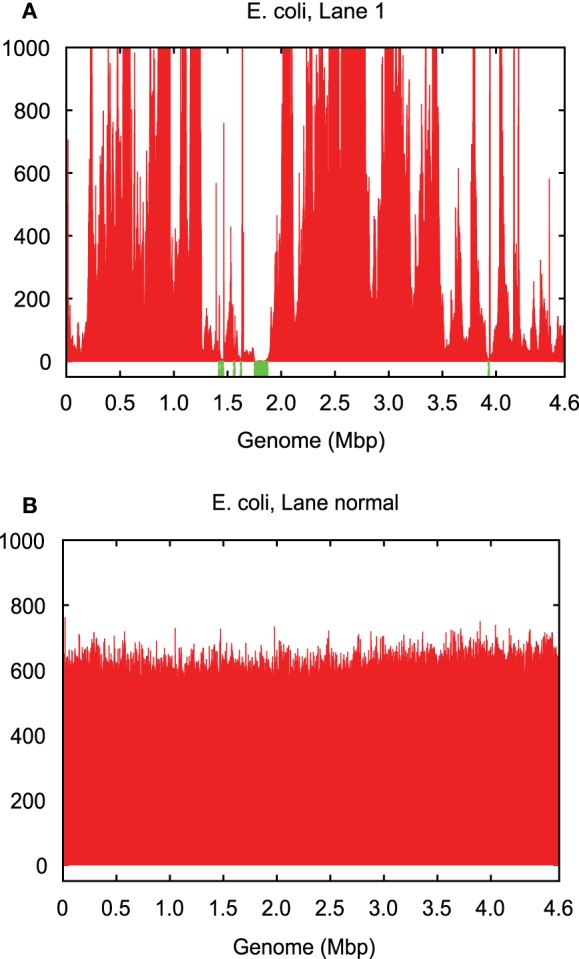
**Genome coverage in (A) single-cell *E. coli* lane 1 vs. (B) normal multicell *E. coli***. Both have an average coverage of ~600×.

Genomes amplified from single cells exhibit highly non-uniform genome coverage and multiple gaps, which are called blackout regions (Chitsaz et al., 2011). For the evaluation of such coverage characteristics in this study, we used amplified DNA originating from two single *Escherichia coli* cells as well as from one single *Staphylococcus aureus* cell (Chitsaz et al., 2011). Although these amplified DNAs were quality checked for preselected genomic loci using quantitative PCR (Rodrigue et al., 2009), they still did not cover the entire genome (Table S1 in Supplementary Material; Figure 2). One single *E. coli* cell was sequenced in four technical replicate lanes (1–4), and the other was sequenced in three technical replicate lanes (6–8) each with a sequencing depth of 600 per lane. The single *S. aureus* cell was sequenced in two technical replicate lanes each with a sequencing depth of 1,800. All nine lanes were sequenced on Illumina GAIIx platform in paired 2–100 bps read mode.

The coverage bias in technical replicates is almost identical, which suggests that the vast majority of bias is caused by MDA. The coverage bias, particularly of the blackout regions, does not always occur at the same genomic loci for different cells of the same genome (Chitsaz et al., 2011). Blackout regions in *E. coli* lanes 1 and 6 sequenced from two independently amplified single cells make up 1.8 and 0.1% of the genome, respectively, but there are no common blackout regions between these two data sets (Table S1 in Supplementary Material). This means that combining the two data sets could fill all gaps and yield a complete genome, which is the property that HyDA exploits with colored coassembly.

### 3.3 Colored Coassembly of *E. coli* and *S. aureus* Mitigates the Effect of Dropout Regions due to Amplification Bias

Single-cell read data sets have highly variable coverage (Raghunathan et al., 2005; Rodrigue et al., 2009) (Table S1 in Supplementary Material; Figure 2), which poses serious challenges for downstream applications such as *de novo* assembly. A number of single-cell assemblers, including EULER + Velvet-SC (Chitsaz et al., 2011), SPAdes (Bankevich et al., 2012), and IDBA-UD (Peng et al., 2012), have been developed to mitigate the adverse effects of non-uniform coverage and maximize the transfer of sequencing information into the final assembly. These efforts have been successful, and the existing single-cell assemblers are able to extract nearly all of the information contained in the input data set. However, the vast majority of single-cell data sets do not encompass the entire genome. We report that combining multiple data sets from the same or closely related species significantly improves the final assembly by filling genome gaps (Table S1 in Supplementary Material). The challenge presented by this method is the subsequent deconvolution of single-cell genomes to avoid chimeric assemblies.

The ideal solution involves the coassembly of multiple data sets without explicitly mixing sequencing reads such that individual assemblies can benefit from the synergy without suffering from chimerism. We propose and implement this solution using the colored de Bruijn graph in HyDA.

We report in Table 1 the coassembly results for six distinct scenarios (Figure S1 in Supplementary Material), each consisting of a combination of the input read data sets: (i) single-cell assembly of *E. coli* lane 1; (ii) single-cell assembly of *E. coli* lane 6; (iii) mixed monochromatic assembly of *E. coli* lanes 1–4 and 6–8, technical replicates of two biologically replicate single cells; (iv) multichromatic coassembly of *E. coli* lanes 1–4 and 6–8; (v) mixed monochromatic assembly of non-identical cells: *E. coli* lanes 1–4 and 6–8, and *S. aureus* lanes 7 and 8; and (vi) multichromatic coassembly of non-identical cells: *E. coli* lanes 1–4 and 6–8, and *S. aureus* lanes 7 and 8, each assigned a unique color. GAGE, a standard genome evaluation tool, which reports the size statistics and number of substitution, indel, and chimeric errors of an assembly, was used to evaluate our assemblies (Salzberg et al., 2012). In all six scenarios, GAGE results (Table 1) comparing the assembly of color 0 with the *E. coli* reference genome are reported. Color 0 corresponds to *E. coli* lane 1 in (i), (iv), and (vi); *E. coli* lane 6 in (ii); and the mixture in (iii) and (v) (Figure S1 in Supplementary Material).

While the state-of-the-art individual single-cell *E. coli* assemblies by SPAdes (SPAdes outperforms IDBA-UD and Euler + Velvet-SC in this case) miss 128,600 (2.77%) and 15,831 (0.34%) base pairs of the reference genome in the two different single cells (Table 3), our coassembly misses only 2,023 (0.04%) of the genome (Table 1), an improvement of 126,577 (2.72%) base pairs of the *E. coli* cell 1. Our coassembly of the two single *E. coli* cells and one *S. aureus* cell misses only 2,136 (0.05%) of the genome. The coassembly algorithm in this work, without any error correction, *k*-mer incrementation, or scaffolding, increases the total assembly size for both *E. coli* lanes 1 and 6 using only the synergy in the input data sets. Our exclusivity ratio (defined below) obtained from the coassembly results completely differentiates *E. coli* and *S. aureus* data sets (Table 2).

**Table 3:** **Evaluation results obtained from GAGE (Salzberg et al., 2012) for assembly of *E. coli* lanes 1 and 6 using E + V-SC (Chitsaz et al., 2011), SPAdes (Bankevich et al., 2012), and IDBA-UD (Peng et al., 2012)**.

Tool	Missing ref. bases (%)	
	Lane 1	Lane 6
E + V-SC	281,060 (6.06%)	109,994 (2.37%)
SPAdes	128,600 (2.77%)	15,831 (0.34%)
IDBA-UD	145,536 (3.14%)	28,583 (0.62%)

### 3.4 Quantification of Similarities and Differences between Colors

Input data sets can be clustered based on the similarity between their assemblies. For a pair of colors *i* and *j*, contigs belonging to both colors are considered shared and contigs belonging to color *i* but not to color *j* are considered exclusive of color *i* with respect to color *j*. We define the exclusivity ratio of color *i* with respect to color *j* as the ratio of the size of exclusive color *i* contigs to the total assembly size of color *i*. The exclusivity ratio for *E. coli* lane 1-lane 6 (Pair 1 in Table 2) is less than 0.5%, while that ratio for *E. coli* and *S. aureus* in the two other pairs (Pair 2 and 3 in Table 2) is greater than 90%. This large difference in exclusivity ratio between Pair 1 and Pairs 2 and 3 is expected in this case, as *E. coli* and *S. aureus* are phylogenetically divergent species belonging to different phyla.

### 3.5 *De Novo* Single-Cell Coassembly of Members of an Alkane-Degrading Methanogenic Consortium

The genomes of 10 cells from three dominant but uncultured bacterial members of a methanogenic consortium (Zengler et al., 1999; Embree et al., 2013), belonging to the families *Syntrophacea* and *Anaerolineaceae* were sequenced from their amplified single-cell whole DNAs: six cells belonging to *Smithella*, two cells belonging to *Anaerolinea*, and two cells belonging to *Syntrophus*. Single cells were isolated from the consortium by fluorescence-activated cell sorting, and the genomes of individual cells were amplified using MDA. MDA products were sequenced using an Illumina GAIIx with 34, 36, or 58 base pair reads. In total, 10 data sets, one per cell, were obtained. The 10 data sets were coassembled with HyDA in a *ten-color* setup, and to exhibit the advantage of the coassembly method, each data set was assembled individually by HyDA. Individual assemblies created by SPAdes and IDBA-UD were used as comparison. The QUAST (Gurevich et al., 2013) length statistics of the resulting assemblies (≥100 bp contigs) are compared in Table 4 and Figures S2–S11 in Supplementary Material. The comparison between individual assembly and coassembly by HyDA demonstrates that coassembly rescues on average 101.4% more total base pairs for all 10 cells (Table S2 in Supplementary Material). Although HyDA does not use advanced assembly features such as variable *k*-mer sizes and paired read information, it can assemble 3.6–54% more total base pairs than both SPAdes and IDBA-UD do in all cells except two cases: *Anaerolinea* F02 and *Smithella* MEK03 (Table 4; Table S2 in Supplementary Material). When all contigs are considered, HyDA coassemblies of *Anaerolinea* F02 and *Smithella* MEK03 are 11% smaller and 41% larger than their SPAdes counterparts, respectively. *Smithella* MEK03 input reads are longer (58 bp) than the reads in some of the other data sets; therefore, the *Smithella* MEK03 assembly contains many short contigs and suffers because of the small *k*-mer size (k = 25) dictated by the shorter reads.

**Table 4:** **Quast (Gurevich et al., 2013) analysis of 10 cells from *Anaerolinea*, *Smithella*, and *Syntrophus* single-cell data sets assembled with HyDA (individual assembly), HyDA (10-color coassembly), SPAdes, and IDBA-UD**.

		*Anaerolinea*		*Smithella*						*Syntrophus*	
		A17	F02	F16	K04	K19	MEB10	MEK03	MEL13	C04	K05
HyDA	Total	54,237	1,278,742	604,769	449,148	371,311	1,182,622	1,666,233	1,150681	252,402	502,469
	N50	**2,935**	8,461	**8,303**	**9,959**	5,416	5,718	6,167	7,315	**5,578**	4,963
HyDA-Cl	Total	**260,386**	1,352,341	**1,323,536**	**720,188**	**840,236**	**1,569,709**	1,945,701	**1,590,259**	**465,091**	**1,265,548**
	N50	850	8,201	6,088	5,239	**7,295**	5,887	5,952	6,977	1,928	3,782
SPAdes	Total	169,413	**1,698,195**	982,263	618,500	653,866	1,514,813	**1,960,722**	1,415,399	390,923	869,586
	N50	1,187	5,944	5,366	9,332	3,834	**8,861**	**11,372**	**10,475**	4,234	3,128
IDBA-UD	Total	144,512	1,441,353	927,009	56,6327	613,399	1,327,742	1,746,656	1,351,465	318,914	804,313
	N50	2,894	**8,756**	3,163	**3,178**	5,751	6,851	8,209	1,0253	4,706	**5,618**

*All statistics are based on contigs of size ≥100 bp. Only those HyDA contigs that have a coverage of at least 1 in the corresponding color are considered. Coverage cutoff was chosen to be 24 for all HyDA assemblies (−c = 24). Total is the total assembly size and N50 is the assembly N50 (the size of the contig, the contigs larger than which cover half of the assembly size). Best result is in bold face*.

### 3.6 Exclusivity Analysis of Ten Assemblies from Single Uncultured Bacterial Cells

Exclusivity analysis revealed that the six Smithella cells clustered into a consistent group as their exclusivity ratios with respect to the two *Anaerolinea* and two *Syntrophus* cells are almost identical (Table 5). It is important to note that *Anaerolinea* A17 and *Syntrophus* C04 assemblies are relatively short, meaning the exclusivity ratios must be interpreted with caution. Although *Syntrophus* K05s exclusivity signature with respect to the six *Smithella* cells is indistinguishable from the six *Smithella* signatures with respect to themselves, the exclusivity ratios of *Syntrophus* K05 with respect to the two *Anaerolinea* cells and *Syntrophus* C04 differentiate *Syntrophus* K05 from the six *Smithella* cells. Slight differences between the *Syntrophus* C04 and K05 exclusivity signatures are not surprising because of the existence of potential intraspecies variations.

**Table 5:** **The exclusivity ratio (%) of row with respect to column for the 10 cells from *Anaerolinea*, *Smithella*, and *Syntrophus* single-cell data sets coassembled using 10 colors with Squeezambler (Taghavi et al., 2013), a tool in the HyDA package**.

		*Anaerolinea*		*Smithella*						*Syntrophus*	
		A17	F02	F16	K04	K19	MEB10	MEK03	MEL13	C04	K05
*Anaerolinea*	A17	0	24	87	95	96	80	82	86	22	19
	F02	77	0	96	98	99	71	68	72	**12**	**5**

*Smithella*	F16	96	96	0	73	73	37	22	38	96	55
	K04	97	97	49	0	67	42	25	45	97	73
	K19	98	98	54	68	0	35	32	32	98	55
	MEB10	96	96	74	48	69	0	24	39	95	57
	MEK03	97	97	73	54	74	38	0	37	96	58
	MEL13	97	97	76	51	68	39	22	0	97	59

*Syntrophus*	C04	44	39	89	96	97	85	86	90	0	64
	K05	77	75	54	76	75	45	41	49	73	0

*Only the contigs of coverage at least 1 in the corresponding color are considered. Coverage cutoff was chosen to be 24 for all HyDA assemblies (−c = 24)*.

### 3.7 Annotation of the *Anaerolinea*, *Smithella*, and *Syntrophus* Assemblies

To assess the quality of coassemblies with HyDA, IDBA-UD, and SPAdes, we used the RAST server to predict the coding sequences and subsystems present in each assembly. The HyDA assemblies are superior to those of SPAdes and IDBA-UD in terms of the number of coding sequences and captured subsystems for one *Anaerolinea*, four *Smithella*, and both *Syntrophus* assemblies (Table 6). For *Smithella* MEB10 and MEK03, the HyDA assembly closely follows the SPAdes assembly, which provides the largest annotation (Table 6). For *Smithella* F16 and *Syntrophus* K05, HyDA assemblies contain significantly more coding sequences (33 and 39%, respectively) and cover more subsystems (29 and 57%, respectively) in comparison to the best of SPAdes and IDBA-UD assemblies.

**Table 6:** **Summary of coding sequences and subsystems predicted by the RAST server (Aziz et al., 2008) for HyDA, IDBA-UD, and SPAdes assemblies of the three alkane-degrading bacterial genomes**.

		HyDA-colored		Spades		IDBA-UD	
		Coding sequence	Subsystem	Coding sequence	Subsystem	Coding sequence	Subsystem
*Anaerolinea*	A17	**212**	8	146	**9**	132	7
	F02	1,283	122	**1,653**	**153**	1,375	121
	F16	**1,197**	**117**	899	91	866	89
	K04	**659**	**89**	559	75	508	66

*Smithella*	K19	**757**	**82**	581	54	572	57
	MEB10	1,491	151	**1,504**	**156**	1,297	138
	MEK03	1,856	180	**1,955**	**200**	1,178	170
	MEL13	**1,535**	**165**	1,435	154	1,384	148

*Syntrophus*	C04	**416**	48	375	**49**	320	36
	K05	**1,216**	**121**	873	68	854	77

*Best result is in bold face*.

To confirm the accuracy of the assemblies, the closest related species to each assembly was computed by the RAST server. For the HyDA, SPAdes, and IDBA-UD *Anaerolinea* F02 assemblies, the closest species was *Anaerolinea thermophila* UNI-1 (GenomeID 926569.3) (no closest genomes data found for *Anaerolinea* A17 by the RAST server). For the HyDA, SPAdes, and IDBA-UD *Smithella* and *Syntrophus* assemblies, the closest species is *Syntrophus aciditrophicus SB* (GenomeIDs 56780.10 and 56780.15). Note that *Syntrophus aciditrophicus SB* is the closest finished genome to the *Smithella* family. This verifies that coassembly does not create chimeric assemblies; otherwise, we would see *Syntrophus aciditrophicus SB* among close neighbors of the *Anaerolinea* assemblies and/or *Anaerolinea thermophila UNI-1* among close neighbors of the *Smithella* and *Syntrophus* assemblies by HyDA.

### 3.8 Metabolic Reconstruction of *Anaerolinea, Smithella*, and *Syntrophus*

Assembly and subsequent annotation of these genomes enables the elucidation of the functional roles of individual, unculturable constituents within the community. *Anaerolinea*, *Syntrophus*, and *Smithella* each represent genera with very few cultured members and only two sequenced genomes – *Anaerolinea thermophila* (no genome paper) and *Syntrophus aciditrophicus* (McInerney et al., 2007) are the only available sequenced genomes from these genera to date. The only member of *Smithella* that has been isolated, *Smithella propionica* (Liu et al., 1999), has not been sequenced yet. In addition to understanding the genetic basis for the unique metabolic capability of this microbial community, the genomes of these particular organisms present an opportunity to explore the breadth of genetic diversity in these elusive genera. Using the advanced genome assembly algorithm, we recently identified the key genes involved in anaerobic metabolism of hexadecane and long-chain fatty acids, such as palmitate, octadecanoate, and tetradecanoate, in *Smithella* (Embree et al., 2013). Based on sequence homology, *Syntrophus* is closely related to Smithella, but we cannot determine if it is also actively degrading hexadecane at this point in time. Only two species of *Anaerolinea* have been isolated and characterized thus far. These species, both isolated from anaerobic sludge reactors, form long, multicellular filaments and are strictly anaerobic (Sekiguchi et al., 2003; Yamada et al., 2006). Each species is capable of growing on a large number of carbon sources, and both isolates produce acetate, lactate, and hydrogen as the main end products of fermentation. Comparison of the Anaerolinea sp. genome derived from single-cell sequencing with the genome of *Anaerolinea thermophila* UN-1 revealed many similarities in potential metabolic capability. The *Anaerolinea* genome obtained from a single cell contains genes for the utilization of galactose and xylose, consistent with a previous physiological characterization of *A. thermophila* (Sekiguchi et al., 2003). Additionally, the single-cell *Anaerolinea sp*. genome encoded for several transporters and genes related to trehalose biosynthesis, suggesting extended metabolic capabilities of this strain. Furthermore, the genome has an extracellular deoxyribonuclease, an enzyme required for catabolism of external DNA, hinting at the strains ability to scavenge deoxyribonucleosides.

## 4 Discussion

We demonstrated the power of genome coassembly of multiple single-cell data sets through significant improvement of the assembly quality in terms of predicted functional elements and length statistics. Coassemblies without any effort to scaffold or close gaps contain significantly more protein coding genes, subsystems, base pairs, and generally longer contigs compared to individual assemblies by the same algorithm as well as the state-of-the-art single-cell assemblers (SPAdes and IDBA-UD). The new algorithm is also able to avoid chimeric assemblies by detecting and separating shared and exclusive pieces of sequence for input data sets. This suggests that in lieu of single-cell assembly, which can lead to failure and loss of the sample or significantly increase sequencing expenses, the coassembly method can hedge against that risk. Our single-cell coassembler HyDA proved the usefulness of the coassembly concept and permitted the study of three bacteria. The improved assembly gave insight into the metabolic capability of these microorganisms, thereby proving a new tool for the study of uncultured microorganisms. Thus, the coassembler can readily be applied to study genomic content and the metabolic capability of microorganisms, and increase our knowledge of the function of cells related to environmental processes as well as human health and disease. The colored de Bruijn graph uses a single *k*-mer size for all input data sets, which has to be chosen based on the minimum read length across all data sets. For instance, Smithella MEK03 input reads are longer (58 bp) than the reads in some of the other data sets, while the Smithella MEK03 assembly contains many short contigs because of the small *k*-mer size (*k* = 25) dictated by the shorter reads. This minor disadvantage can be remedied by using advanced assembly features such as variable *k*-mer size, alignment of reads back to the graph and threading, and utilization of paired-end information.

## Author Contributions

NM carried out genome assembly and evaluation, helped with metabolic reconstruction analysis, participated in development of HyDA, and drafted the manuscript. ME and HN participated in acquisition of the alkane-degrading consortium genomic data and drafted the manuscript. KZ participated in the project conception, participated in acquisition of the alkane-degrading consortium genomic data, and drafted the manuscript. HC participated in the project conception, developed HyDA, carried out interpretation of results, and drafted the manuscript.

## Conflict of Interest Statement

The authors declare that the research was conducted in the absence of any commercial or financial relationships that could be construed as a potential conflict of interest.
