# Supplementary material for: Efficient Synergistic Single-Cell Genome Assembly
Source: Front Bioeng Biotechnol. 2016 May 23;4:42. doi: 10.3389/fbioe.2016.00042 (PMC4876485; doi:10.3389/fbioe.2016.00042)
Supplement: Supplementary file 1 [file presentation_1.pdf]

Supplementary Material: Efficient synergistic single-cell genome assembly

Narjes S. Movahedi, Mallory Embree, Harish Nagarajan, Karsten Zengler, and Hamidreza Chitsaz*

*Correspondence:
Hamidreza Chitsaz
chitsaz@chitsazlab.org

SUPPLEMENTARY TABLES

Table S1. Evaluation of blackout regions. The number, mean length, N50, and total size (percentage) of blackout regions in the *E. coli* and *S. aureus* data sets as reported previously (Chitsaz et al., 2011). All lanes are *E. coli* except the one marked *S. aureus*.

Data set	No. of blackouts	Mean length	N50	Total (%)
lane 1	94	1220	5558	84K (1.8%)
lane 6	50	193	518	5K (0.1%)
lanes 1 and 6	0	0	0	0 (0.0%)
S. aureus	2	95	83	143 (0.0%)
**Replicates of *E. coli* lane 1:**				
lane 2	91	1183	4700	77K (1.7%)
lane 3	92	1159	5842	77K (1.7%)
lane 4	88	1225	6156	76K (1.7%)
**Replicates of *E. coli* lane 6:**				
lane 7	63	153	456	5K (0.1%)
lane 8	61	185	573	6K (0.1%)

Table S2. Improvement achieved by colored HyDA. Improvement of colored HyDA in comparison with individual HyDA and the best single-cell assembler.

		Colored HyDA improvement versus	
		Individual HyDA %	Best Result 2 %
Anaerolinea	A17	380	**53**
	F02	5	-19
Smithella	F16	119	**35**
	K04	60	**17**
	K19	126	29
	MEB10	32	4
	MEK03	16	-0.7
	MEL13	38	12
Syntrophus	C04	85	**19**
	K05	153	46

SUPPLEMENTARY FIGURES

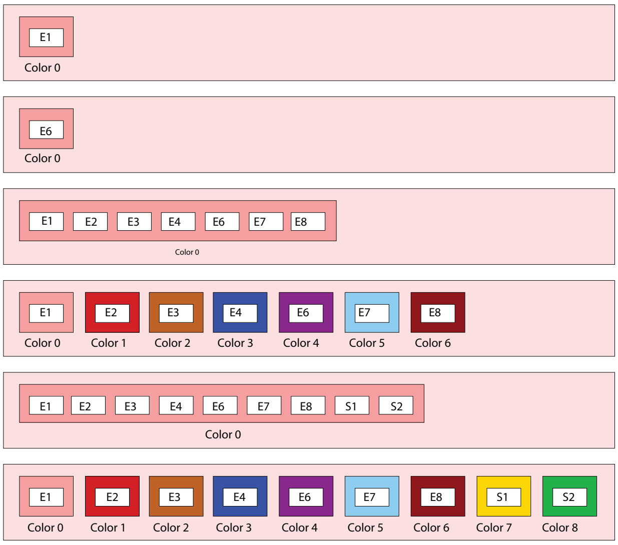

Figure S1. The six assembly scenarios: (i) single-cell assembly of *E. coli* lane 1; (ii) single-cell assembly of *E. coli* lane 6; (iii) mixed monochromatic assembly of *E. coli* lanes 1-4 and 6-8, technical replicates of two biologically replicate single cells; (iv) multichromatic co-assembly of *E. coli* lanes 1-4 and 6-8; (v) mixed monochromatic assembly of non-identical cells: *E. coli* lanes 1-4 and 6-8 and *S. aureus* lanes 7,8; (vi) multichromatic co-assembly of non-identical cells: *E. coli* lanes 1-4 and 6-8 and *S. aureus* lanes 7,8, each assigned a unique color.

REFERENCES

Chitsaz, H., Yee-Greenbaum, J. L., Tesler, G., Lombardo, M.-J., Dupont, C. L., Badger, J. H., et al. (2011). Efficient de novo assembly of single-cell bacterial genomes from short-read data sets. *Nature Biotech* 29, 915–921

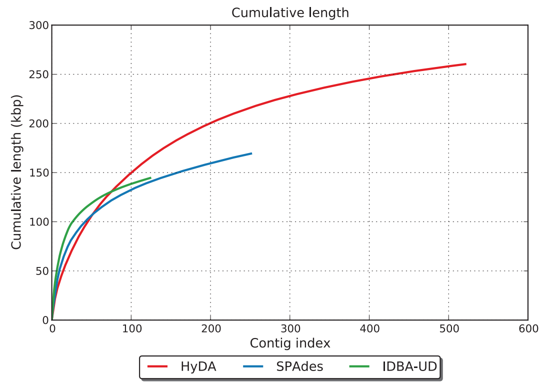

Figure S2. QUAST comparison plots: HyDA, SPAdes, and IDBA-UD assemblies of *Anaerolinea* A17.

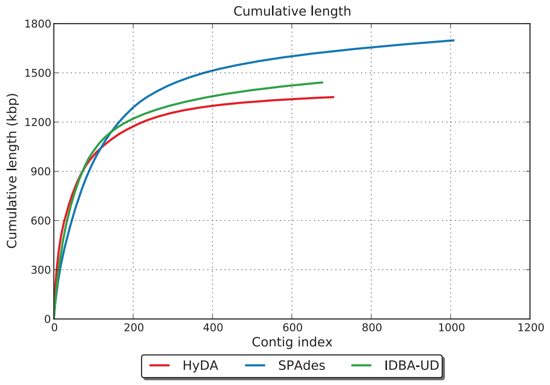

Figure S3. QUAST comparison plots: HyDA, SPAdes, and IDBA-UD assemblies of *Anaerolinea* F02.

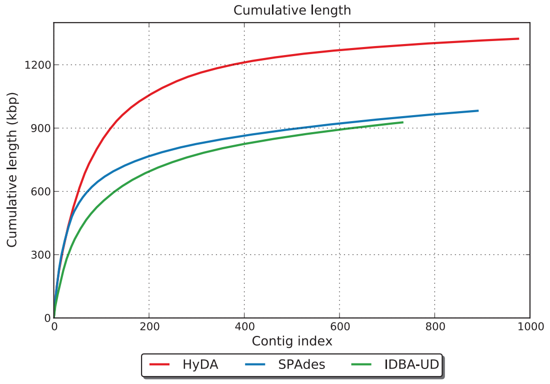

Figure S4. QUAST comparison plots: HyDA, SPAdes, and IDBA-UD assemblies of *Smithella* F16.

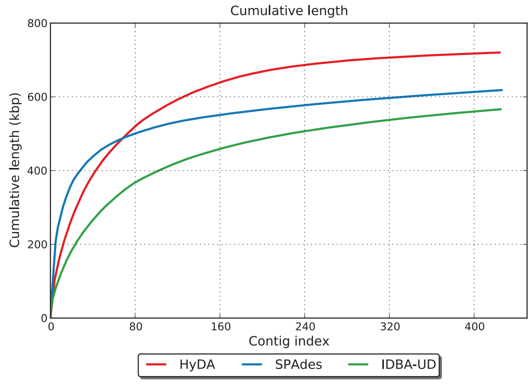

Figure S5. QUAST comparison plots: HyDA, SPAdes, and IDBA-UD assemblies of *Smithella* K04.

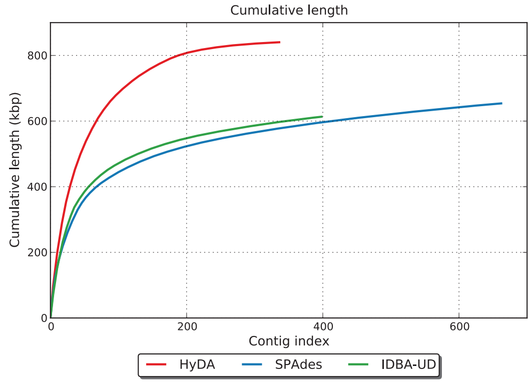

Figure S6. QUAST comparison plots: HyDA, SPAdes, and IDBA-UD assemblies of *Smithella* K19.

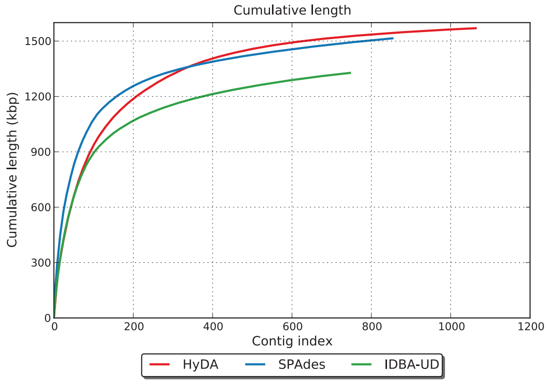

Figure S7. QUAST comparison plots: HyDA, SPAdes, and IDBA-UD assemblies of *Smithella* MEB10.

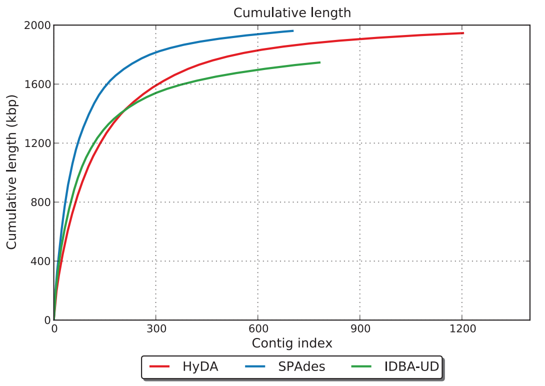

Figure S8. QUAST comparison plots: HyDA, SPAdes, and IDBA-UD assemblies of *Smithella* MEK03.

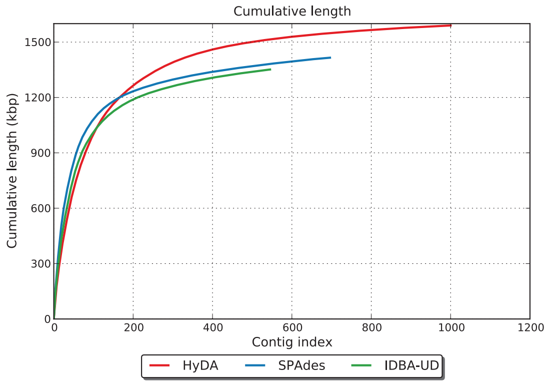

Figure S9. QUAST comparison plots: HyDA, SPAdes, and IDBA-UD assemblies of *Smithella* MEL13.

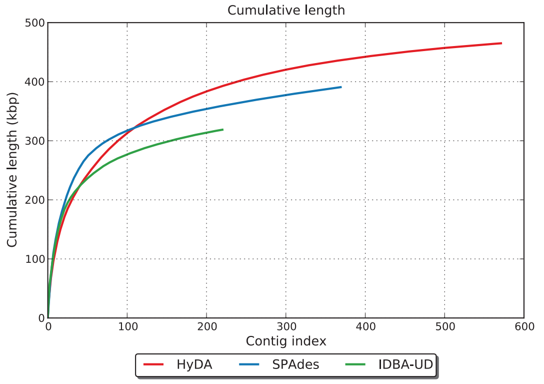

Figure S10. QUAST comparison plots: HyDA, SPAdes, and IDBA-UD assemblies of *Syntrophus* C04.

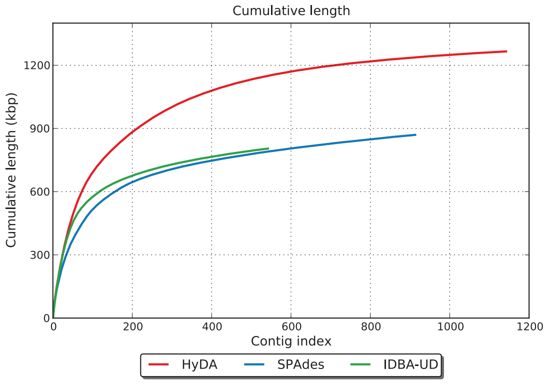

Figure S11. QUAST comparison plots: HyDA, SPAdes, and IDBA-UD assemblies of *Syntrophus* K05.
